# Spatio-temporal analysis of sheep and goat pox outbreaks in Uganda during 2011–2022

**DOI:** 10.1186/s12917-023-03788-w

**Published:** 2023-10-28

**Authors:** Gerald Nizeyimana, Patrick Vudriko, Joseph Erume, Frank Mubiru, Wilfred Eneku, Savino Biryomumaisho, Robert Mwebe, Eugene Arinaitwe, Rose Ademun, Stella Atim, Chrisostom Ayebazibwe, Dennis Muhanguzi, Robert Tweyongyere

**Affiliations:** 1 Department of Pharmacy Clinical and Comparative Medicine, School of Veterinary Medicine and Animal Resources, College of Veterinary Medicine Animal Resources and Biosecurity, Makerere University, Kampala, Uganda; 2 Department of Bimolecular and Bio Lab Sciences, School of Biosecurity, Biotechnology and Laboratory Sciences, College of Veterinary Medicine Animal Resources and Biosecurity, Makerere University, Kampala, Uganda; 3 Emergency Centre for Transboundary Animal Diseases, Food and Agriculture Organisation of the United Nations, Kampala, Uganda; 4 National Animal Disease Diagnostics and Epidemiology Centre, Directorate of Animal Resources, Ministry of Agriculture, Animal Industry and Fisheries, Entebbe, Uganda

**Keywords:** Sheep pox virus [SPPV], Goat pox virus [GTPV], Sheep and goat pox [SGP], Passive surveillance, Active surveillance, Sheep and goat pox, Reporting, SGP antibodies

## Abstract

**Background:**

Sheep and goat pox (SGP) caused by sheep poxvirus (SPV) and goat poxvirus (GPV) respectively; are transboundary and World Organisation for Animal Health (WOAH)-notifiable viral diseases. There is barely any coherent information about the distribution and prevalence of SGP for Uganda. We therefore conducted this study to describe the temporal and spatial distribution of SGP suspected outbreaks in Uganda for the period 2011–2020 as well as serologically confirm presence of SGP antibodies in suspected SGP outbreaks reported in 2021–2022.

**Results:**

Thirty-seven [37] SGP outbreaks were reported across the country during the study period. North-eastern region [that comprises of Karamoja region] had the highest number of outbreaks [*n* = 17, 45%]; followed by Central [*n* = 9, 2.4%], Northern [*n* = 8, 2.2%] and Western region [*n* = 3, 0.08%]. Reports from district veterinary personnel indicate that the prevalence of; and mortality rate and case fatality rate associated with SGP were 0.06%, 0.02% and 32% respectively. There was a steady increase in the number of reported SGP outbreaks [x̄ = 4] over the study period. Seropositivity of SGPV antibodies in outbreak sheep and goats that were investigated during the study period [2021–2022] was [*n* = 41, 27%, 95 CI;]

**Conclusion:**

Our analyses of SGPV passive and active reports indicate that SGP is present in Uganda with a decade long average of four outbreaks per annum. During this period, about a third of all SGPV-clinically infected animals died. SPG is therefore a major constraint to small ruminant health and productivity in Uganda. Introduction of animals from infected herds and breach in farm biosecurity were the most important predictors of SGP outbreaks. In addition to the already existing SGP commercial vaccines, small ruminant screening for SGPV before introducing them to naïve herds and ensuring on farm biosecurity should be part of the SGP control tool pack for Ugandan small ruminant farmers.

**Supplementary Information:**

The online version contains supplementary material available at 10.1186/s12917-023-03788-w.

## Background

Sheep and goat pox [SGP]; a disease caused by closely related pox virus strains [Genus Capri poxvirus; Family Poxviridae], is an economically important contagious disease of small ruminants in Africa above the equator [including Uganda], Middle East and western Asia [1–3]. SGP contributes to the bane of poverty in these regions, where about 330 million poverty-stricken people reside majority of whom are women, children and the elderly [4–6]. For most of the households in SGP endemic countries, small ruminants are a direct source of food [of milk, meat, and meat products], fiber, wool, and cash resources. SGP-associated losses are due to mortalities, reduced small ruminant production and productivity, costs incurred on treatment and prevention [antibiotics and vaccines] as well as incumbrances to international trade in small ruminant and small ruminants products trade [1, 7–10].

SGP presents with high fever, generalized macules that progressively become papules or skin necrotic lesions [11]. The disease presents with post-mortem nodular lesions of internal organs [11, 12]. Morbidity due to SGP varies greatly [1- 90%] with animal breed and endemic status; with imported breeds and naïve flocks being the most susceptible [13]. Young and naïve flocks suffer the highest case fatality rate [of up 100%] [14]. SGP is spread directly and indirectly through contact with infected animals, aerosols of nasal secretions, infected saliva and dried scabs, fomites and transportation vehicles [15]. SGP has no curative treatment. It is only prevented by immunopropylaxis and biosecurity [1].

Like any other trans-boundary animal diseases, SGP requires strong passive and active surveillance system with effective zoo-sanitary disease control measures [13, 16]. This is however not always feasible in resource [financial and human]-constrained countries. As a result, SGP becomes endemic in such countries for example Uganda. This is even in the face of compelling evidence on the profitability of SGP vaccination among subsistence farmers [10]. When effectively implemented, SGP vaccination is highly effective with positive economic returns [10].

However, SGP control, and surveillance efforts in Uganda are still negligible. This is, in part, characterised and aggravated by very scanty passive and active SGP surveillance and disease epidemiology data sets that are very essential for informing risk-based SGP control efforts. This study was therefore undertaken to partially fill this literature gap but more importantly to generate SGP spatial temporal datasets that are essential for designing and implementing an SGP risk-based control [for example targeted vaccination] program. We described SGP spatial temporal distribution patterns from district self-reported suspected SGP outbreaks [for the years 2011–2020] and confirmed that suspected outbreaks during the years 2021and 2022 were associated with SGPV infections based on antibody detection using double Capripox multispecies antigen enzyme linked immunosorbent assay [DCMA-ELISA].

## Results

### Temporal [2011–2020] patterns and reporting levels of suspected SGP cases in Uganda

**Fig. 1:**
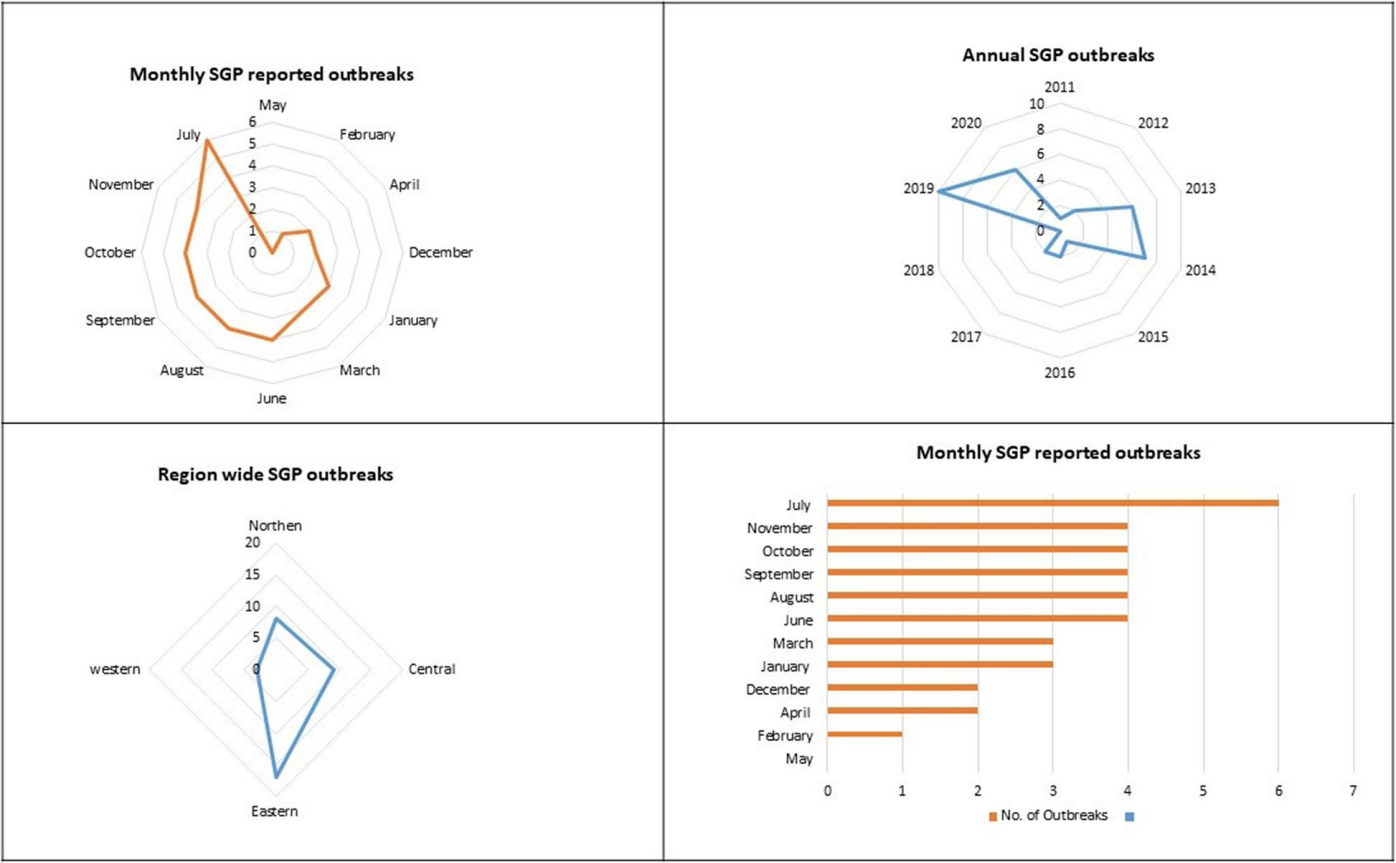
Temporal patterns of SGP suspected outbreaks [2011–2020]

A small population [1,269/2,161,953, 0.06%] of sheep and goats was suspected to be SGP clinical cases over the study period [2011–2020]. About a third [405/1,269, CFR 32%] of all SGP suspected cases died over study period with an average mortality rate of 0.02% [405/2,161,953]. Only [4,071, 37.3%] of all the expected reports [*N* = 11,792] were turned in by the district veterinary staff to the epidemiology unit of the National Diseases Diagnostics and Epidemiology centre (NADDEC) over the study period (Table 1). Month and regional wise analysis of the reporting patterns indicated that July had the highest number of reported outbreaks while May had the lowest number of outbreaks over the study period (Fig. 1).

**Table 1:** Suspected SGP outbreak reports and level of reporting during 2011 – 2020

Year	No. suspected outbreaks	Reporting districts [= n]	Reports expected [= N]	Reports submitted [= n]	% Reporting level n/N*100	Susceptible population [a]	Suspect cases[b]	Deaths [c]	% Morbidity [b/a*100]	%MR [c/a*100]	% CFR [c/b*110]
2011	1	81	972	103	10.6	1,500	12	7	0.80	0.47	58.3
2012	2	103	1232	772	62.7	180	44	4	24.4	2.22	9.09
2013	6	112	1344	808	60.1	52,790	201	37	0.38	0.07	18.4
2014	7	112	1344	870	72.5	52,882	175	75	0.33	0.14	42.9
2015	1	112	1344	567	42.2	9	4	0	44.4	0.00	0.00
2016	2	112	1344	287	21.4	140,057	55	10	0.04	0.01	18.2
2017	2	112	1344	538	40	147	14	0	9.52	0.00	0.00
2018	0	115	1380	268	19.4	0	0	0	0.00	0.00	0.00
2019	10	115	1380	299	21.7	964,388	526	163	0.05	0.02	31.0
2020	6	133	1596	367	23	950,000	238	109	0.03	0.01	45.8
**Total**	**37**		**11,792**	**4,071**	**37.36**	**2,161,953**	**1269**	**405**	**0.06**	**0.02**	**31.9**

*MR* Mortality rate, *CFR* Case fatality rate, * Multiply. Obtained from National Diseases Diagnostics and Epidemiology centre (NADDEC) epidemiology unit archives

### Spatial distribution [2011–2022] of suspected SGP outbreaks in Uganda

**Fig. 2:**
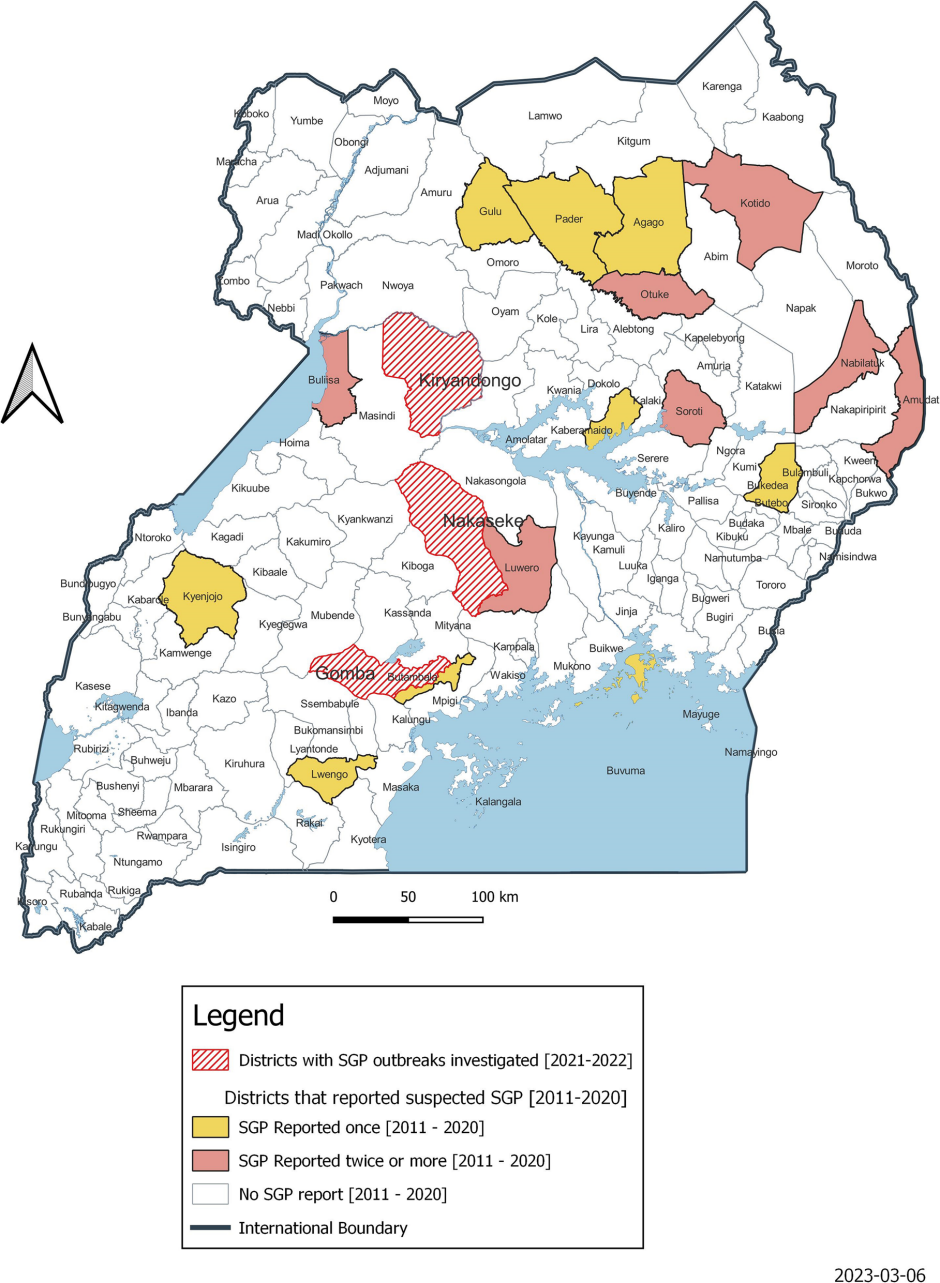
Spatial distribution [2011–2022] of suspected SGP outbreaks in Uganda

A total of thirty-seven (37) suspected SGP outbreaks occurred during the study period. These suspected SGP outbreaks were reported in 15 districts from four regions of the country namely, north-eastern eastern Uganda [17/37; 45%], Central [9/37; 24%], Northern [8/37; 22%], and western (3/37; 0.08%]. At least one suspected outbreak was reported for each of the study years except 2018: a year with the lowest disease reporting level by the district personnel (Table 1, Fig. 2).

Goat herds had majority [22/37; 59.5%] of the suspected outbreaks compared to mixed goat and sheep herds [15/37; 40.5%]. However, majority of the suspected cases [823/1269; 64.9%] were from mixed goat and sheep herds while 35% of the cases [446/1,269] were reported from herds comprised of only goats. We were unable to segregate deaths by species in mixed flocks due to the data quality that we worked with. However, highest mortalities [269/405; 66.4%] occurred in mixed goats and sheep herds compared to those that occurred in herd that comprised of only goats [136/405; 33.6%].

### Clinical manifestation of sheep and goat pox cases

**Fig. 3:**
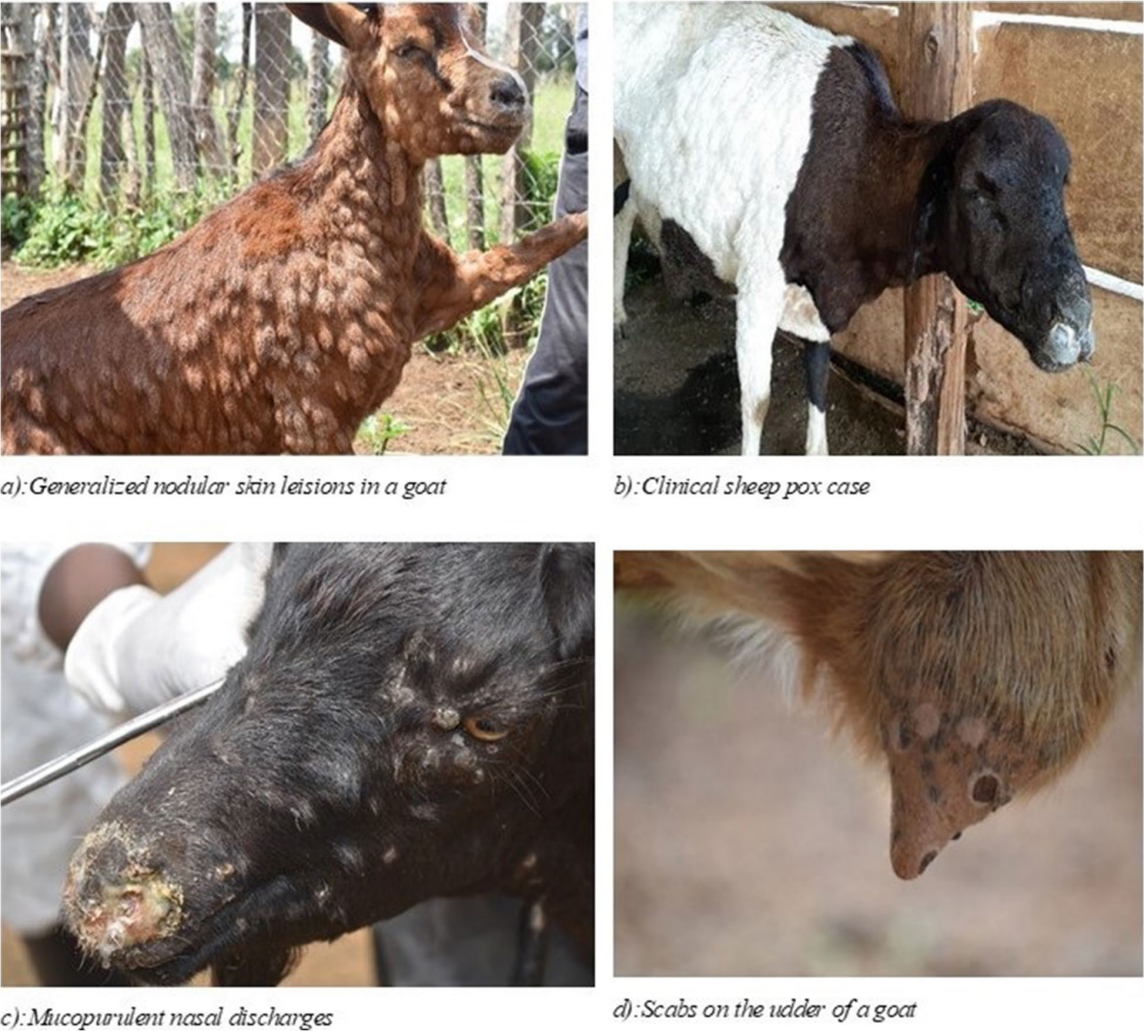
Suspected clinical cases of SGP from outbreaks investigated

**Fig. 4:**
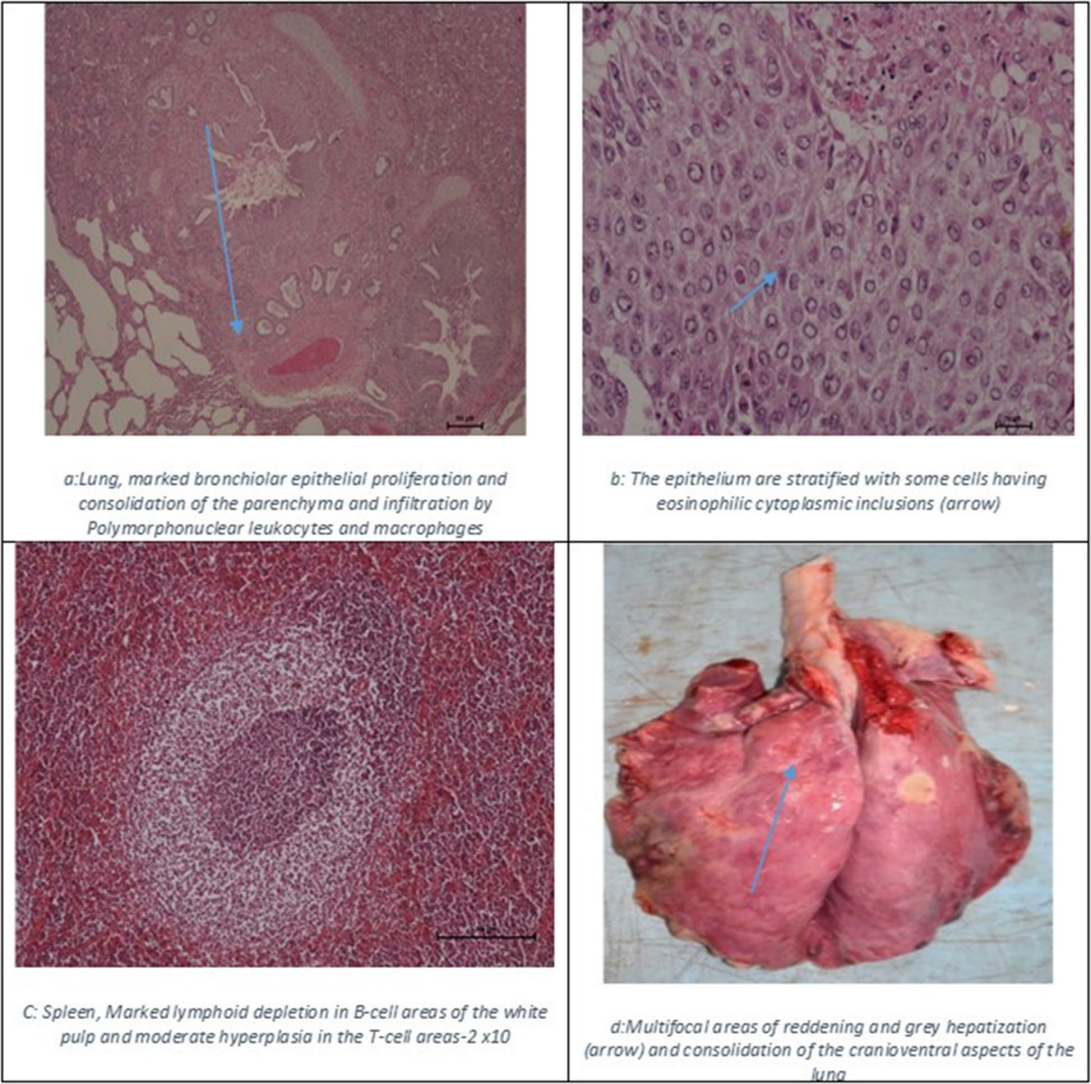
Necropsy and histopathological lesions of suspected SGP case

Suspected clinical cases presented the typical SGP signs that included; fever ranging from 39.5 degrees to 41 degrees, generalized skin nodular lesions, enlarged peripheral lympnodes, and mucopurulent nasal discharges (Fig. 3). At necropsy, we noted acute fibrinous pleuropneumonia, necrotizing rhinitis, multifocal skin scabs, enlarged mediastinal lympnodes, remarkable fibrin deposits at the thoracic inlet and cranial ventral aspects of the lung with reddening and consolidation of the portions while histopathological examination showed remarkable lesions in the spleen and lungs as shown in (Fig. 4).

### Confirmation of SGP antibodies from suspected outbreak farms during [2021–2022]

About 27% [41/166] of all animals sampled from suspected SGP outbreak farms during the years 2021/2022 were positive for SGP antibodies. Most of positive animals had recently recovered from the disease with most of their lesions resolved (Table 2).

**Table 2:** SGP seropositivity descriptors on outbreak farms investigated during 2021–2022

Suspected SGP descriptors	No. of investigated farms [a]	No. sampled	No. with SGP signs [b]	Period since onset of clinical signs [c]	No. dead [d]	Mortality rate [%; d/a^a^100]	Case fatality rate [%; d/c^a^100]	N [%;n/N^a^100] seropositivity
1**. Location [District]**								
i. Nakaseke	435	91	265	[16–21]	93	21.38	35.1	31.9 [29]
ii. Kiryandongo	460	36	150	15	50	10.87	33.3	27.8 [10]
iii. Gomba	410	39	105	10	5	1.22	4.76	5.1 3 [2]
**Overall**	**1305**	**166**	**520**	[10–21]	**148**	**11.2**	**24.4**	**27 [CI:95]**
2**. Herd of origin**								
i. SGPF1	150	30	80	16	40	26.7	50.0	36.7 [11]
ii. SGPF 2	70	16	50	18	20	28.6	40.0	81.3 [13]
iii. SGPF 3	75	9	45	21	30	40.0	66.7	33.3 [3]
iv. SGPF 4	460	36	150	15	50	10.9	33.3	27.8 [10]
iv. SGPF 5	140	36	90	16	3	2.1	3.33	5.56 [2]
iv. SGPF 6	410	39	105	10	5	1.2	4.76	5.23 [2]
**3. Breed**								
i. Mubende goats	150	30	80	16	40	26.7	50.0	36.7 [11]
ii. Admixtures goats	1101	131	435	[16–21]	108	9.8	24.8	22.9 [30]
iii. Ankole fat tailed sheep	54	5	5	10	0	0.00	0.00	0.00 [0]
**4.Smallruminant category**								
i. Sheep	54	5	5	10	0	0.00	0.00	0.00 [0]
ii. Goats	1251	161	515	15–21	148	11.8	28.7	25.5 [41]

^a^Admixture goats were cross breeds of Boer and Mubende goat

Antibodies were detected at each of the farms investigated. More seropositive animals were detected at SGPF [2, 1, 4 & 3]. Out of the 6 farms, only one had both sheep and goats.

## Discussion

Based on passive surveillance data, only 16 districts reported 37 SGP suspected outbreaks during the period: 2011 to 2020. None of the suspected outbreak reports was further investigated and confirmed, although the clinical presentation reported indicated that SGP is prevalent in the various regions of Uganda. Consistent with previous reports [17, 18] there was seasonal variation in the number of SGP outbreaks reported during the study period with the majority of the outbreaks reported during the dry season [June–August] [19]. Dry seasons are associated with maximum small ruminant flock comingling and disease transmission due to increased animal movement in search of pasture and water [20, 21]. This is particularly so in transhumant pastoral communities of north-eastern Uganda [Karamoja region] when there is periodic uncontrolled influx of ruminant flocks from neighbouring countries as well as inter district animal movements during the dry seasons in search of water and pasture [19]. Additionally, dry seasons are associated with animal stress [nutritional and physical] and therefore increased susceptibility to disease [22]. For the same reason, and as previously reported [14], SGP outbreaks were associated with higher mortality [up to 40%] and case fatality rates [up to 65%] according to farmers’ verbal autopsy reports [14].

Reporting levels of 37.3% would indicate that SGP just like any other transboundary animal diseases is heavily under reported in Uganda [23, 24]. This alongside the low sensitivity of passive surveillance system, limited resources and incentives for front line animal health officers to report and or deliver public services further compounds the problem [24]. Effective control of SGP would therefore require heightened passive and active surveillance to generate data that are requisite to designing and implementing a risk-based disease control programs for example by [vaccination and regulated animal movements] [25]. All suspected SGP outbreaks were neither confirmed [by December 2020] by World Organisation of Animal Health (WOAH)-recommended diagnostic techniques nor were they reported through WOAH animal health information system [26] as should be the norm for all notifiable animal diseases [25]. This is likely to perpetuate insidious disease transmission; a status quo that needs to change.

We confirm [clinically and serologically] herein, for the very first time, that SGPV is circulating in small ruminant populations in Uganda. Clinical cases presented with typical clinical signs of SGP infections (see Fig. 3) namely; fever [up to 41.0 ^0^C], generalized skin nodular lesions [papules, macules, nodules and dry scabs] and respiratory involvement characterized by dyspnoea, mucoid and mucopurulent nasal discharges [3, 14]. Investigated suspected SGP outbreaks were associated low seroconversion rates consistent with a previous report [13] that has indicated that SGPV antibody titres are optimal within 2–3 weeks of onset of clinical signs requiring that outbreaks are investigated during this period. We were unable to determine the actual time when SGP clinical signs were first observed in investigated flocks because none of the flocks was managed by a professional veterinarian and relied on farmers recall capacity in absence of records. It is therefore likely that we took samples outside the SGP optimal sampling time a period when, detectable neutralizing antibodies are low, explaining low levels [27%] of seroconversion rates in the investigated outbreak small ruminant herds [27]. Additionally, panic sales that are triggered by suspected SGP outbreaks could have contributed to low seropositivity in investigated outbreak flocks as it was unlikely to find index cases at the farm. Serodiagnosis however, presents challenges with SGP diagnosis due to the high clinical and serological relationship between sheep and goat pox that make species differentiation difficult even at molecular level, more so in East Africa where there is lack of host specificity among SGP viruses and inability to differentiate vaccinated from infected [2, 28, 29].

We observed poor biosecurity measures at outbreak farms including poor carcass disposal, mixing of sick and apparently healthy animals, uncontrolled movement of animals and people on farms, limited disease knowledge, lack of disinfection and no record of screening animals for SGP before purchase. Consistent with previous reports, these practices were positively correlated with SGP seropositivity on investigated cases [27]. The farmers managed clinical cases with a broad range of antibiotics such as [Oxytetracycline Penicillin and multivitamins] consistent with previous report [9].

In this manuscript, we were unable to characterise the virus strains and their phylogenetic relationships to those previously reported globally. Additionally, we did not investigate the drivers of these outbreaks over time. These data, if available, would improve the diseases epidemiology in the region. Future studies are therefore recommended to fill these rather important literature gaps.

## Conclusion

Our analyses of SGPV passive and active reports indicate that SGP is present in Uganda with a decade long average of four outbreaks per annum. During this period, about a third of all SGPV-clinically infected animals died while about a third [27%] of all suspected cases of SGP that were reported during the period 2021–2022 were confirmed to be SGPV seropositive. SGP is therefore a major constraint to small ruminant health and productivity in Uganda. Introduction of animals from infected herds and breach in farm biosecurity were the most important predictors of SGP outbreaks. In addition to the already existing SGP commercial vaccines, small ruminant screening for SGPV before introducing them to naïve herds and ensuring farm biosecurity should be part of the SGP control tool pack for Ugandan small ruminant farmers.

## Materials and methods

### Study area and population

Uganda is an African country in East Africa crossed by the equator with altitude ranging from 1000–1400 m, and moderately warm climate with temperature ranging from 20 -25 degrees and mean annual rainfall of 900 to 1500 mm [30]. Uganda is endowed with abundant savannah grass and woodlands favourable for goats and sheep production. Uganda’s total goat and sheep population stands at 16.9 million and 4.6 million, respectively [31] and has been growing at 3.1% and 2.8%, respectively per annum [32]. Goats and sheep production systems are predominantly extensive with very few shoats’ intensive farms. In Uganda up to 95% of the goat and sheep breeds are indigenous comprising of the small East African, Mubende and Kigezi goats [33], while for sheep up to 99.2% are indigenous and 0.8% exotic [34].

### Study design

This was a cross-sectional study involving analysis of passive surveillance data of suspected sheep and goat pox records reported by districts to National Animal Diseases Diagnosis and Epidemiology Centre [NADDEC] [January 2011- December 2020]. The reports were accessed with permission from the chief veterinary officer, Ministry of agriculture animal industry and fisheries, Uganda. This was in addition to an active investigation of four suspected SGP outbreak cases reported in three districts in Central Uganda in the year 2021 and 2022.

### Passive sheep and goat pox surveillance data

Monthly passive surveillance reports submitted to (NADDEC) is mandatory for all districts in Uganda. Monthly surveillance data submitted [January 2011 and December 2020] was retrieved from the database, examined for completeness and all reported suspected case events of SGP disease were documented as reported SGP outbreaks for the purpose of this study. Suspected outbreak data was employed to portray and describe the temporal and spatial distribution of sheep and goat pox in Uganda.

### Sheep and goat pox outbreak investigation

Four suspected SGP outbreaks reported [2021 and 2022] were actively investigated by physical, clinical examination of cases and laboratory examination of samples for SGP neutralising antibodies using ELISA. The four outbreaks were in the districts of Nakaseke [where four farms were involved], Kiryandongo and Gomba where one farm in each of the two districts was involved. For the purpose of this study, investigated farms were code named SGPF1, SGPF2, SGPF3, SGPF4, SGPF5 and SGPF6 in order of investigation. Farmers specifically reported to area veterinary officers a strange disease in goats and sheep characterized by generalised nodular skin swellings with high morbidity that was likened to lumpy skin disease in cattle. All the farms practiced extensive production system where the flocks were housed overnight and grazed by free range in designated paddocked savannah grass and woodlands. All the farms investigated reported no history of vaccination against sheep and goat pox. Five out six farms had a history of recent introduction of goats in the last one month. Of the six farms, only one had both goats and sheep. On each of the farms investigated, ten (10) goats with clinical signs were randomly picked at the farm for clinical examination [rectal temperature, examination of the integumentary, lymphatic system, alimentary and the respiratory systems]. One goat from Nakaseke district (SGPF1) with pox clinical signs was purchased and euthanized using xylazine and ketamine followed by carotid artery laceration for a systematic post-mortem and histopathology examination. Blood samples [serum] were collected from selected suspected cases and processed for laboratory analysis. The collected blood samples were transported in cool box to NADDEC and stored at -20 °C until the analysis.

### Laboratory assay

The sera were tested for antibodies against sheep and goat poxvirus using a double Capripox antigen multispecies ELISA Test Kit test (ID Screen®, ID vet, Garbles, France). *Briefly; *50 μL of each test serum sample were thawed, diluted in 50 μL of dilution buffer 19 and added to an ELISA plate coated with Capripoxvirus purified antigen. Positive and negative control sera were similarly diluted and added to the designated wells on each ELISA plate. The ELISA plate was incubated for 90 min at room temperature, wells emptied and washed 5 times with wash solution. On to the wells, 100 μL of conjugate was added and incubated for 30 min at room temperature. Wells emptied and washed 5 times followed by addition of 100 μL per well of substrate and the plate incubated in the dark at room temperature for 15 min. This was followed by addition of 100 μL per well of stop solution and the optical density (OD) read at 450 nm using a microplate reader (Biochrom Asys UVM 340, Cambridge, United Kingdom). For each sample; percentage positivity was calculated as optical density of sample minus optical density of negative control divided by optical density positive control minus optical density negative control multiplied by 100, represented in formula below as;

$$\frac{\mathrm{S}}{\mathrm{P}}\mathrm{ \%}=\frac{\mathrm{ODsample}-\mathrm{ODNc}}{\mathrm{ODPC}-\mathrm{ODNC}}\mathrm{ X}100$$

Test samples with S/P percentage of less than 30% were considered negative and those with S/P percentage greater of equal to 30% were taken as positive.

Tissues for histopathology were formalin fixed, paraffin embedded and the slides processed by routine haematoxylin and eosin staining procedure. The slides were examined and micrographs taken from a microscope (Nikon Eclipse Ci, Japan) with mounted camera.

## Data analysis

Raw data from National Disease Diagnostics and Epidemiology Centre [NADDEC] database of suspected sheep and goat pox reported between [January 2011 to December 2020] was cleaned and exported into Microsoft excel spreadsheets for management and generation of descriptive statistics. Descriptive statistics included; morbidity rate, mortality rate and case fatality rates. The SGP outbreaks spatial distribution over the 10-year period was generated using QGIS version 3.18, to portray the distribution and location of reported suspected outbreaks.

True antibody seropositivity was determined based on the apparent seropositivity [AP] adjusted for the sensitivity [Se] (91%) and specificity [Sp] [99.7%] of the ELISA test Kit. seropositivity was determined by dividing the total positive samples to the number sampled. The true antibody seropositivity was used to calculate farm level based prevalence based on the formula by Stevenson, 2007 [35]; $[\mathrm{True \,antibody \,seropositivity }=\mathrm{ AP}+\mathrm{SP}-1/\mathrm{ Se}+\mathrm{SP}-1]$.

### Supplementary Information

**Additional file 1:** **Supplementary file 1.** Shows a table of suspected sheep and goat pox outbreaks from districts that reported, including calculations of estimated morbidity, mortality and case fatality rates based on cases, sick, death and population risk in areas that reported.

## Data Availability

All data and materials are available except the national sheep and goat Pox data set that requires prior approval of the chief veterinary officer of Uganda, Ministry of agriculture Animal Industry and Fisheries Uganda, with the file accessed from Dr. Mwebe Robert (rmwebe@gmail.com), the National Epidemiologist.

## Abbreviations

CFR: Case fatality rate
DCMA-ELISA: Double Capripox Multispecies Antigen Enzyme Linked Immunosorbent Assay
ELISA: Enzyme linked immunosorbent assay
FAO: Food and Agriculture Organisation
GTPV: Goat Pox Virus
NADDEC: National animal disease diagnostics and epidemiology centre
ODNC: Optical Density Positive control
ODPC: Optical Density Negative Control
Se: Sensitivity
SGP: Sheep and Goat Pox
SGPF1-6: Sheep goat pox outbreak farm 1–6
Sp: Specificity
SPPV: Sheep Pox Virus
UIA: Uganda Investment Authority
WOAH: World Organisation for Animal Health
