# Supplementary material for: Spatio-temporal analysis of sheep and goat pox outbreaks in Uganda during 2011–2022
Source: BMC Vet Res. 2023 Oct 28;19:224. doi: 10.1186/s12917-023-03788-w (PMC10612334; doi:10.1186/s12917-023-03788-w)
Supplement: Supplementary file 1 — Additional file 1: Supplementary file 1. Shows a table of suspected sheep and goat pox outbreaks from districts that reported, including calculations of estimated morbidity, mortality and case fatality rates based on cases, sick, death and population risk in areas that reported. [file 12917_2023_3788_MOESM1_ESM.docx]

**Additional supplementary File 1**

| ***Year*** | ***District*** | ***No of cases*** | ***No of animals*** | ***Prevalence*** |
| --- | --- | --- | --- | --- |
| 2011 | Amudat | 12 | 1500 | 0.8 |
| 2012 | Gulu | 31 | 180 | 17.2 |
| 2012 | Soroti | 13 | N/A* | N/A* |
| 2013 | Kotido | 7 | 61 | 11.5 |
| 2013 | Soroti | 6 | 45 | 13.3 |
| 2013 | Agago | 19 | 54 | 35.2 |
| 2013 | Otuke | 32 | 130 | 24.6 |
| 2013 | Otuke | 125 | 500 | 25.0 |
| 2013 | Amudat | 12 | 52000 | 0.0 |
| 2014 | Otuke | 50 | 500 | 10.0 |
| 2014 | Otuke | 38 | 122 | 31.1 |
| 2014 | Bulisa | 21 | 47 | 44.7 |
| 2014 | Amudat | 5 | 52000 | 0.0 |
| 2014 | Otuke | 14 | 28 | 50.0 |
| 2014 | Bulisa | 28 | 100 | 28.0 |
| 2014 | Kaberamaido | 19 | 85 | 22.4 |
| 2015 | Kyonjojo | 4 | 9 | 44.4 |
| 2016 | Lwengo | 50 | 140000 | 0.0 |
| 2016 | Butambala | 5 | 57 | 8.8 |
| 2017 | Bukedea | 2 | 19 | 10.5 |
| 2017 | Pader | 12 | 128 | 9.4 |
| 2018 | N/A* | N/A* | N/A* | N/A* |
| 2019 | Luwero | 140 | 220000 | 0.1 |
| 2019 | Nabilatuk | 15 | 300 | 5.0 |
| 2019 | Luwero | 90 | 220000 | 0.04 |
| 2019 | Nabilatuk | 10 | 600 | 1.7 |
| 2019 | Kotido | 11 | 2932 | 0.4 |
| 2019 | Luwero | 116 | 260000 | 0.04 |
| 2019 | Nabilatuk | 1 | 125 | 0.8 |
| 2019 | Nabilatuk | 30 | 400 | 7.5 |
| 2019 | Buvuma | 23 | 31 | 74.2 |
| 2019 | Luwero | 90 | 260000 | 0.03 |
| 2020 | Luwero | 100 | 210000 | 0.05 |
| 2020 | Amudat | 13 | 120000 | 0.010833 |
| 2020 | Amudat | 7 | 120000 | 0.005833 |
| 2020 | Amudat | 12 | 120000 | 0.01 |
| 2020 | Amudat | 16 | 120000 | 0.013333 |
| 2020 | Luwero | 90 | 260000 | 0.034615 |
|  |  | **1269** | **2161953** | **0.06** |

Legend:

**Additional supplementary File 1:** Shows a table of suspected sheep and goat pox outbreaks from districts that reported, including calculations of estimated morbidity, mortality and case fatality rates based on cases, sick, death and population risk in areas that reported
